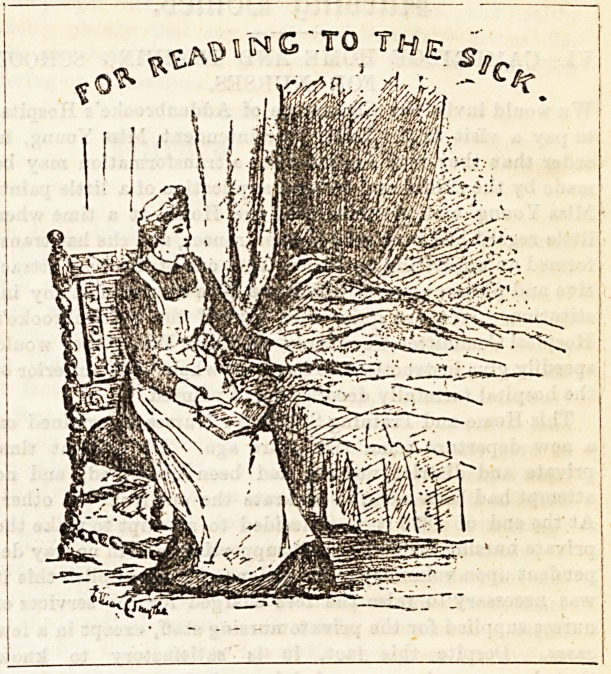# The Hospital Nursing Supplement

**Published:** 1892-08-20

**Authors:** 


### "The Hospital" Nursing Mirror.

Being the Extra Nursing Supplement of "The Hospital" Newspaper.

Contributions for this Supplement should be addressed to the Editor, The Hospital, 140, Strand, London, W.C., and should have the word "Nursing" plainly written in left-hand top corner of the envelope.

### En Passant.

THE ABERDEEN DISTRICT NURSING ASSOCIATION.—This association has had thirty-nine cases under Miss Armstrong's care since she began work on May 20th, and already the committee have decided on engaging a second nurse who has been trained at Aberdeen Royal Infirmary, and who, on the completion of her training in the Central Home in Edinburgh, will go to work under Miss Armstrong. The Duchess of Fife has consented to become the President of the Association.

LADY STUDENTS AT THE EDINBURGH ROYAL INFIRMARY. — Having resolved to admit lady students to the Edinburgh Royal Infirmary, the question now presents itself as to how the scheme is to be worked. It has been arranged that two wards shall be set apart for the use of the twenty students proposed to be sent by the Scottish Association for the Medical Education of Women. These wards will each hold twenty patients, and are on the ground floor of the surgical department; they will be used exclusively by women students, one ward being reserved for surgical, the other for medical cases. The legal enactment that eighty beds are necessary before a diploma can be obtained has been overcome by an arrangement to permit the lady students to attend the eye, ear, and skin wards. So for the present the difficulties are solved.

NOT LOST SIGHT OF.—At some of our hospitals it is easy enough for those who have been trained there to keep in touch with their old "Alma Mater" in more ways than one, and it is a great mistake which many make to think the old associations are no good and that they don't matter. For instance, at the "London" each departing nurse is given a paper on which it is stated that "nurses holding the London Hospital certificate of training and working elsewhere, may, if they keep the Matron fully informed of their subsequent appointments, have particulars concerning them entered in a supplementary register kept for that purpose. This arrangement has been made with a view to giving nurses the full benefit of their past connection with the hospital in the way of references, and in obtaining any future work they may desire." This is an excellent plan, as by this means nurses can always identify themselves.

ISLE OF MAN NURSES.—The efforts of the Committee, which was formed towards the close of last year for the purpose of providing funds to supply a nurse for the sick-poor in the Isle of Man, have met with a full measure of success. The Committee of Noble's General Hospital have now been enabled to arrange with the Matron that the work of visiting the sick-poor shall be performed by one of the skilled nurses of the hospital acting in rotation, and the Matron has undertaken the responsibility of supervising; they have also gone a step farther, recognising the fact that not only the sick-poor, but those who are not poor, cannot always have their wants in respect of nurses readily supplied, and have engaged two nurses, who are placed under the Matron's supervision, and who, when not engaged by private individuals, are to assist in the hospital or in attending to the outside sick-poor, as the Matron may direct. It is satisfactory to know that, in consequence of the constant demand for the private nurses, the hospital has incurred no additional expense through its public-spirited effort to supply an evidently great want.

DISTRICT NURSES AND THE POOR LAW.—We have often been asked lately if we could at all gauge the effect of the recent provision of the Local Government Board in allowing Guardians to provide the nursing of their out-relief poor. The memorandum pointed out that the person who fills this office should have thorough practical training in the nursing of the sick. From many inquiries we fear that Boards of Guardians as a whole have not jumped at the permission given to them. There was a letter last week in the *Hampshire Advertiser*, signed "Looker On," commenting adversely on the fact that the poor law Guardians of Southampton had refused Dr. Maclean's suggestion that district nurses should be employed in the nursing of out-door paupers. In the same week, at Darlington, it was suggested that the Board should join the Nursing Association of the district and so obtain trained nurses whenever they require them. Whether they have done so we have not yet learned, and we fear that the Southampton decision only too closely resembles that of most other Boards of Guardians, and that the scheme is somewhat of a failure. Those of us who have witnessed personally the sufferings of the sick poor know what a large field of good the Guardians by their permission really have before them, and we wonder at the inhumanity which has kept hundreds of Boards from utilising the power given to them when the need of so many out-paupers could be relieved by an infinitesimal amount of trouble and money.

"MANNERS MAKYTH MAN."—At clubs, on the Stock Exchange, in the streets, the chimney-pot hat has become a familiar and, possibly, an appropriate covering for the male head in the nineteenth century; but we fail to understand the reason why it should be considered suitable for wearing during a walk through a hospital ward. We note with pleasure the habit which working men have of removing hats or caps when they visit their sick friends; in fact, we should now be inclined to question the ward management where this courtesy is omitted. We should be justified in criticising the general tone of a place where men, seated at the bedside, keep their heads covered habitually. We have observed Royal Princes, aye, and a King, too, removing their hats ere they entered the abode of the suffering poor, and this implied respect and kindly courtesy are swiftly recognized and appreciated. A little child was one day heard to say, "The Prince of Wales took 'is 'at off when 'e come 'ere to see we, but that there committee gentleman allers sticks to hisn." "Yes," rejoined the lad in the next bed, "I guess 'e ain't much to look at wi'out 'is 'at; he fancies 'isself, he do! When he gits talkin' to our Sister, and don't even offer to off wi' 'is 'at, I wonder 'er don't offer to mind it for 'im, while he lords it up and down the place. I wish 'er would, don't you, Jim?" Probably the same idea, though differently expressed, has passed through the mind of many a nurse and ward sister, who endeavours to maintain "courtesy to all men," and feels herself more heavily handicapped in her gently firm discipline than need be the case, if those who ought to know better set a practical example in direct opposition to her theories. When corridors are draughty, we can sympathise with the necessity for covering up an indifferently thatched scalp, but surely no such need should arise in the patients' quarters. The ventilation must be very unscientifically managed if observable blasts of air gain admittance, and most decidedly no room can be suitable for the housing of the sick if a casual caller is driven by cold to walk about with his hat on!

### The Management of Consumptive Patients.

### III.—GENERAL ADVICE.

Many of the preceding rules and regulations could only be carried through with the patient under the immediate observation of the nurse; their efficiency depends upon her vigilance, tact, and power of control. If the nurse faithfully performs her duty with regard to the expectoration and the ventilation, it is reasonable to suppose that at the end of three or four weeks' stay in an institution conducted on the lines laid down a patient will perforce have become saturated to a certain extent with these new ideas, and will, in a measure, carry them into effect when at home. Besides these special injunctions, there is still much general advice, which, if properly tendered, cannot fail to be of use to the consumptive. The advisability of marriage among patients with consumption claims our attention. Unfortunately, it is no unusual sight to see young men and young women with consumption of well marked type coming into our chest hospitals in order to improve in health so that they may marry as soon as they leave the institution. Frequently, on visiting days, the lovers of patients in the hospital will come and boldly ask the doctor how soon he thinks such and such an one will be fit to marry; they seem to be ignorant or careless of the fact that by entering into the married state with consumptives they are exposing themselves to a fearful risk. Take a case as an example. A young girl with consumption of moderate severity becomes engaged, and before long, marries; her disease does not trouble her for some time, in fact, she feels better than usual; her health seems to improve rather than become worse; in due time she bears two children, but now her cough has increased. She loses a considerable amount of flesh, her disease hurries on at a dreadful pace, and she dies. But the record of misery does not end here. Her children, one after another, fall victims to the same disease, and her husband, once so strong and healthy, now begins to show unmistakable signs of the same complaint. Here the lives of three people were irretrievably wrecked by the simple want of forethought and advice.

It is, undoubtedly, the fact that a large proportion of those having consumptive parents, become themselves consumptive at some period of their existence, so that the nurse should use all her powers of reasoning and persuasion to drive out of her patient's head any idea of marriage; in doing this she will be performing her duty, but in the present state of society, we fear her exertions in this direction will be attended with only partial success; the public have yet to understand that the idea of marriage with a person with unsound lungs should be as foreign to their natures as the idea of marriage with a person of unsound mind. The patient should be advised as to his way of living; he should, when walking out, carry a pocket spittoon, into which he can expectorate; the spittoon can be fitted into an inside pocket, previously lined with waterproof. In this way the dangerous practice of spitting on the floors of railway carriages and tramcars, in the streets, and public places can always be avoided. When in his home he should keep the windows continually open to allow of large quantities of fresh air. No one else should sleep in the same room with him, and the bed-room window should be open all night.

The consumptive should, as far as possible, choose an occupation where there is a chance of being out in the open air, and one in which there is little or no dust. If he has choice of localities, a high-lying, dry district is the one in which he will be most likely to battle successfully with his disease. There yet remains to treat of the consumptive patient's exercise.

There are few diseases which require so little of treatment by confinement to bed as consumption, except during and after an attack of hæmoptysis, or an exacerbation of the disease, as manifested by continued high fever, or some other grave complication; the consumptive patient will be more likely to improve if allowed to be up and about a certain part of the day, than if kept rigidly in bed. His spirits are kept up, he regards himself less as a man ill, and he improves consequently; whereas, if he be allowed to lie in bed he frequently relapses into a listless, moping condition, and often in hospitals, at least, a consumptive taking to bed is a sign of approaching end. So unless there is some reason to the contrary, a person with consumption should be coaxed to get up and about as much as possible. Patients who can be got up should be sent out into the open air whenever practicable; every opportunity should be seized upon for gentle exercise out of doors. The only two atmospheric conditions against this practice are rain or snow, or damp weather. In the depth of winter, with the frost and snow on the ground, the consumptive may, with greatest prospect of benefit to himself, take exercise in the open air; so also in the summer heat he may with impunity spend the greater part of the day out. This is the secret of the improvement taking place in so many consumptives who are sent to health resorts; the weather there is so warm and fine that, in spite of themselves, they spend the day outside. There is nothing special in the air, except that it admits of their being continually in the open.

To recapitulate then, the main facts in these papers: There are excellent reasons for believing (1) that consumption can be spread by means of the expectoration; (2) that there is little danger of this expectoration giving consumption to others unless it be allowed to become dry; (3) that consumptive patients should always expectorate into a spittoon with some disinfecting solution in it; (4) that consumptives require as much pure, fresh air as can be supplied to them, whether by ventilation or by exercise in the open air; (5) that consumptive people should not marry.

By the inculcation of the rules and principles here laid down, by the exhibition of tact, forbearance, and determination to her patient, the nurse may do something to help those with consumption to improve, and much to prevent others from becoming its victims.

### Death in Our Ranks.

Many of our readers knew personally, and many others will have heard of, the work of Miss Emily Minet, Superintendent of the Stratford-on-Avon Nursing Home, whose death we have to record with much regret. Miss Minet died on Monday, August 8th, at Stratford, aged 57, and was buried on the 11th at the cemetery, many of her friends being present. Beautiful flowers had been sent in great numbers, amongst them being a water lily wreath from the local branch of the G. F. S., and flowers from the nurses, servants, and patients at the home. Miss Minet undertook the superintendence of the Nursing Home in 1872 when it was started, and by careful just management she won the love and co-operation of all her many friends and fellow-workers, and for twenty years she carried on her work till her health broke down. Her sympathies were very wide, and anything that tended to diminish the sufferings of the poor won her ready help; her loss will be felt very keenly by all who knew her.

Female Medical Students are not carrying everything before them at the Johns Hopkins Hospital. It is said that a few of the trustees are still opposed to the admission of women students to the new parts of the hospital. Considering that the trustees accepted 110,300 dollars from the women of the eastern cities, it will be interesting to watch their antics if they try to wriggle out of what is to all intents a promise.

### The Nightingale Fund.

REPORT FOR 1891.

On the 31st of December last, there were 13 special and 19 nurse probationers in the school at St. Thomas's Hospital. During 1891 thirty probationer nurses were entered on the register of certified nurses after completing their year of training, and seventy nurses received the £2 gratuity, which is given for three years to those who have completed a year's service satisfactorily in some good hospital or institution for the benefit of the sick poor. Certainly "Nightingales" never lack appointments. During the past year twenty-eight who completed their training received the following appointments: Three as nurses and three as ward Sisters, one as assistant night Superintendent, three as night nurses, four as day nurses, and twenty-three as extra nurses all at St. Thomas's Hospital; one as night Superintendent at the Liverpool Workhouse Infirmary, one as nurse at the Sheffield Public Hospital, one as nurse at the Radcliffe, Oxford, one as day nurse and one as night nurse at St. Marylebone Infirmary.

This week we have news of a Nightingale nurse who is going away to Fiji. It is a great comfort to find that, however far away the post, or however lonely or arduous, there is always somebody found ready to volunteer and go out and fill it.

Miss Gordon reports that the probationers have given satisfaction in their work in the wards—the basis and most important part of their training—and the instruction given to them by the ward Sisters and nurses had been "efficient and well attended to;" and how much the future of these young nurses depends on efficient practical instruction it is impossible to gauge. Miss Crossland, the Home Sister, has, as usual, been indefatigable in teaching the probationers in class and in helping them to prepare for and understand the Professor's lectures, and also in caring for their good discipline, comfort and happiness in the Home.

During the past year the appointment of Dr. Bristowe and Mr. Croft, lecturers to the school, ceased by effluxion of time. Dr. Bristowe gave eleven medical lectures and five clinical, and Mr. Croft lectured on anatomical and surgical matters, and gave five lectures of a clinical character as well; while Dr. Bernays (whose death at the beginning of this year was a great loss to the school) lectured on heat, water, air, and diet. We wish diet lectures and instruction held the place in some of our other hospitals, both London and provincial, which they ought to have. The greatest ignorance as to the most elementary points in the culinary art is noticeable in many of our nurses, and a superficial knowledge of the necessary components of foods which is worse than useless.

Miss Vincent, the Matron of St. Marylebone Infirmary, Notting Hill, in her report to the Council on the training school there, states that, with twelve probationers in the infirmary on January 1st, twenty-three new candidates had been admitted during the year, twelve had completed their year's training and received appointments on the Infirmary staff, and twelve had left before completing the year's training, leaving eleven in the Home on December 31st. During the year twelve nurses received their first and thirteen their second gratuities.

The whole expenses for the year for the Nightingale school, including the contribution to expenses of training school at St. Marylebone New Infirmary, were £2,149 14s. 3d.

---

"So Providence for us, high, infinite,
Makes our necessities its watchful task,
Hearkens to all our prayers, helps all our wants,
And e'en if it denies what seems our right,
Either denies because t'would have us ask,
Or seems but to deny, and in denying grants."

REAL KINDNESS.

Poor human nature is loath to believe that anything apparently severe can be kind, so that we rebel against afflictions instead of taking them to heart, and trying to find out in what way we can improve; we thus let the opportunity slip, and then require a second, or it may be a third or fourth blow, to drive the lesson home. And yet we all know how necessary it is to direct anything that has life in order to prevent its going wrong. The higher and nobler the creature, the easier and better it is to manage. The lion, the elephant, and the horse can each be tamed and made to submit to man's control, and be content under it, but the fierce and cruel tiger can never be tamed, his brutal spirit bursts out again and again, even when he is caught and trained from his birth, while the ass and the swine, with their stubborn perversity fight and struggle to the end, determined, seemingly, to make their own lives and their master's a burden.

Man is not free from this universal law. The sweetest and most loving child needs correction, even though it almost breaks our hearts to use the chastening rod. In God's sight we are but children, taking our own way, doing silly, foolish things, or worse, out of thoughtlessness or sheer perversity. It grieves our heavenly Father to see us despise good and turn to evil, so in love He bends our will by His afflictions.

An illness prevents our going to some entertainment where we should meet bad companions who would lead us astray, or a disappointment which crushes our happiness for a time, may be the saving of body and soul in this world and the next. As an instance of this, a young and pretty girl was engaged to be married to an officer, and expected in a short time to go with him to India to join his regiment there. A few weeks before the wedding he played her false, and married another and a richer wife. Strange to say, the ship in which the newly-wedded couple embarked was run down in the Channel, and every passenger on board perished. The poor, deceived girl, in her grief and misery, was led to see that God had saved her, perhaps from an unhappy union, certainly from a sudden and awful death. In this we recognize how a loving Parent worked for His child's good, but we cannot always see so clearly. The Almighty has many lessons to teach His own, their faith often requires strengthening; then He hides His operations from them, and they must submit and wait in patience till He vouchsafes light on their path. Let us then with noble courage accept our lot, and kiss the hand of Him Who doeth all things well, Who often hides a blessing behind a frowning face.

### Nursing Homes.

### VI.—CAMBRIDGE HOME AND TRAINING SCHOOL FOR NURSES.

We would invite the Committee of Addenbrooke's Hospital to pay a visit to the Lady Superintendent, Miss Young, in order that they may realise what a transformation may be made by the skilful and tasteful application of a little paint. Miss Young took possession of the Home at a time when little regard had been paid to appearances, and she has transformed an uninviting apartment into one of the most attractive and pleasant rooms which we have ever seen at any institution. We are quite sure that if the Addenbrooke's Hospital Committee would only visit Miss Young they would speedily give instructions to have the whole of the interior of the hospital tastefully decorated and painted.

This Home and Training School for Nurses determined on a new departure some two years ago. Up to that time private and district nursing had been combined, and no attempt had been made to separate the one from the other. At the end of 1890 it was decided to attempt to make the private nursing institution self-supporting, and in no way dependent upon voluntary subscriptions. To accomplish this it was necessary to raise the fees charged for the services of nurses supplied for the private nursing staff, except in a few cases. Despite this fact, it is satisfactory to know that the system has succeeded beyond the highest expectations of its promoters, and that, as the result of the year's working, after the payment of the whole of the expenses, amounting to nearly £1,100, there was a balance at the bank of £73. No doubt Miss Young's excellent management has had much to do with this triumph, for knowing the difficulties attending the introduction of a new system, however sound it may be in principle, we cannot regard it as anything less. The terms charged for nurses are £1 11s. 6d. per week in ordinary cases, or two guineas per week if the nurse sleeps at the Home. Mental and infectious cases are charged for at the rate of two guineas per week, and the fee for one day or one night is 7s. 6d. The nurse's laundry and travelling expenses have to be paid by the employer, who is further charged one guinea for disinfection in fever cases. Twenty-two private nurses are at present engaged, who attended 300 cases during 1891, three of which were nursed gratuitously, and eight at reduced fees. Two probationers are at present in training.

The District Nursing Branch is entirely supported by voluntary contributions, and provides efficient and gratuitous nursing for the poor in their own homes. Nursing appliances and necessary clothing and bedding are lent by the Lady Superintendent. Infectious cases, those more suitable for removal to the hospital, and those requiring parish relief and skilled nursing are not undertaken. Miss Sayle, who was the Superintendent of this branch up to August, 1891, displayed great energy and efficiency in organising the district nursing in its present condition. The present Superintendent is Miss Kyneston, who is well-known in Cambridge, and was formerly night Superintendent of Addenbrooke's Hospital. Five district nurses and two midwives are engaged in the work. During last year the former attended 393 cases and paid 13,657 visits, exclusive of those of the Superintendent, and the former attended 250 cases out of 270, a fee of 5s. being paid by each maternity case to the funds of the district nursing. For the purposes of the work, Cambridge has been divided into six districts, and anyone requiring a nurse's services has to apply to the district nurse of the locality where she resides, or to the Lady Superintendent. The Committee have wisely determined to permit the district nurses to give instruction to a limited number of pupils in their rounds, for which a payment of one guinea for a course of twelve lessons has to be made. The receipts during 1891 amounted to £880, against an expenditure of a little over £475, the latter including £20 for food, nursing appliances, and chemist, which shows that the bodily necessities of the patients are not overlooked.

Altogether, we have formed a very high opinion of the quality of the work which is being done by the Cambridge Home and Training School for Nurses, and we would advise anyone interested, or the representatives of any district where it is proposed to start a district nurse, to visit Cambridge with a view to ascertaining how this useful branch of nursing can be done inexpensively and thoroughly. No doubt the Committee are to be congratulated upon having secured the services of Miss Young, who is an exceptionally able administrator and has an unusual aptitude for the work, but it must not be forgotten that both Miss Young and Miss Kyneston were both trained at Cambridge, and that Addenbrooke's has therefore good cause to be proud of the results obtained by its school for nurses.

### Why Probationers Break Down.

Now that everybody has at least one relation, or intimate friend, engaged in nursing work, somewhere or other, no general conversation can be indulged in, for any length of time, without the subject of hospitals coming under discussion, and it is very instructive as well as interesting to listen to the different views held on the subject.

As it is generally the newest probationer who has most suggestions to make, respecting improved ward management, so it is usually the lady who has never done a day's work in her life, who authoritatively commends or denounces the career which has been adopted by her friend. Whilst one person thinks it must be "so awfully trying to have to make a poor, dirty creature clean," another says "it must be so sweet to look after those dear little children," and both are right, whilst both are wrong!

But there is always one part of the subject on which there exists unanimity of opinion, "So many girls break down, become perfect wrecks, in fact!" As this is asserted by most people who, although not personally acquainted with hospital life, have yet seen the effect of it on the aforesaid relations and friends, it seems worth while to attach some importance to the statement. First of all, as regards the "broken down" candidates (for, mark you, it is very seldom that a fully-trained and qualified nurse is thus quoted), are they injured in health by any real, honest work done by them, or is it not very often the case, that they are victims to some old infirmity which the unusual strain of the exciting new life amidst unaccustomed sights and scenes, combined with extra fatigue, has developed anew? Probably, had the fact of the original weakness been confided to the matron, she would never have accepted the candidate, and mutual disappointment would have been avoided.

Again, there are many girls who take to nursing in order to divert their thoughts from some great loss—the death of a dear one, or the faithlessness of a lover, or a general dissatisfaction with quiet home-life—and at first the novelty of the fresh surroundings, new interests, and constant employment, answer the desired end. The girl feels the keen edge of her own trouble becoming blunted; she is insensibly drawn away from the sad or bitter reflections which formerly held possession of her mind, and she finds with surprise that she is regaining her spirits, and taking an interest in her own future, instead of feeling that her life was already "played out." But with this reaction a stage is soon reached where the fact that nursing is not her vocation evinces itself. Having taken up the work merely from an idea that it might act as an opiate on her sufferings, she finds, as soon as she regains her normal mental condition, that she has made a grave blunder. If she has means of her own, or if her parents are willing to welcome her back, the difficulty ends here, for she soon disappears from the hospital horizon. But two other things may occur, the first being a discovery that the life thus unwittingly adopted has become a most attractive one. She feels each day, as the great systematic nature of the work unfolds itself before her, that she is drawn more and more strongly towards it, she sees the nobleness of serving others, of striving to aid in the cure of the injured or diseased bodies of the suffering poor around her; she learns daily lessons of patience, endurance, and cheerfulness from their unconscious example, and she feels that she is her right place at last, though truly more by accident than design.

The second thing which may occur in the case we are supposing, is that the girl, awakened to the fact that she has blundered, sees no way to retrieve the mistake; she is too proud to own, to those who could help her, this failure on her part, a variety of circumstances combine to hinder her from seeking any other path, and so it seems easier, in every way, to let herself drift on. She remains, with no heart in her work, and but little hope for the future, and by-and-by the discontent of her mind reacts on her body, she grows weary and takes no trouble to fight against what she calls her "fate." She does not sleep well, and her appetite fails, for she does not relish the plain food with which a healthy probationer is thoroughly satisfied. Presently she "breaks down," she either catches some infectious disease, or else is found to be simply "run down," and is obliged to go off duty, and perhaps this is the best thing that can happen to her, if it enables her to retire altogether from an uncongenial sphere.

Of course, there are other kinds of girls who take to hospital work simply for amusement, or from wilfulness, &c., and it is by no means unusual for one of this class of young ladies to give herself invalid airs directly she begins to feel the strain of continuous employment, and, after a day's visit to her sympathetic friends, she returns, armed with a letter, with which she, next morning, trots off to headquarters. This is found to contain the decided opinion of her parents that she "is not nearly strong enough for hospital work, and that the family doctor (quite an intimate old friend, has known her from a baby!) says she must leave at once, or her health will be ruined!" This matter is generally easy of arrangement, as the services of such a person are of little value anywhere. Occasionally, a victim to hysteria, whose friends are told by some medical man that regular occupation is desirable for her, gets put into a training school, and whether this is quite fair towards patients or her fellow nurses, although open to discussion, does not concern us here; but should the poor girl "break down" under the ordeal, surely hospital life ought not to receive the blame!

Any young nurse who neglects to take proper care of herself, who treats all warnings on the subject of health with contempt, thinking it meritorious to continue on duty when she is not fit to be at work, is acting not only foolishly but very selfishly. To escape what she calls "making a fuss," in other words, to avoid the momentary annoyance of bringing the subject of her own health into prominence, and thereby putting the care of it into others' power, she will go on, to the last limit of her over-taxed strength, thus entailing anxiety and serious trouble on those who are responsible for her. Yes; there is often more selfishness displayed in this way than many of us would care to own and illnesses whose onset is thus neglected become very serious indeed when it is too late for them to be either arrested or treated with the comparative ease possible only if the mischief be detected at its commencement. So, avoiding undue anxiety or fussiness, we beseech all probationers and nurses to take fit and proper care of that best of all possessions, good health, realising plainly that any neglect of this duty brings discredit on their hospital, grief on their friends, and much suffering on themselves.

### Nurses' Bookshelf.

### "THE NAULAHKA."*

This is a book we can cordially recommend to nurses wanting to be wholesomely amused and interested. The sketch of the girl who dedicates herself to the service of Indian women is excellently drawn, it is perfectly and consistently harmonious throughout. Mr. Kipling's scenes from life in Hindustan are too familiar to need praise —in fact he writes "knowledgeably," and we already feel that he has taught us more of that wonderful land than any previous writer. But we should dearly love to know whether it is Mr. Wolcott Balestier's inexperience which gives to Kate such an anomalous position as is inferred by her practising as a doctor after only two years of training? We English think three years are needed to evolve a thoroughly skilled nurse, and five are now necessary for the curriculum through which a lady must pass ere she becomes a qualified medical woman. Certainly America is a rapid country, but this is rather too swift a progress for even a daughter of the Stars and Stripes.

### ESSENTIALS OF MEDICAL ELECTRICITY.†

This manual presents evidence of a great deal of very thorough work on the part of the compilers, Dr. Stewart and Dr. Lawrence. We believe the little volume will prove of considerable value to medical students of both sexes, and also to those trained nurses who are wishful to study the scientific as well as the practical points of electricity. It is not a work for probationers to study in their early days, but we can strongly recommend it to the advanced student or to the young practitioner, in whose early career there often comes leisure for study and observation, which he will strive in vain to secure in later times, when his days seem all too short for the serious demands made by his professional engagements. In the book before us we find the writers have taken some pains to show the forms in which electricity exists, as well as giving us an account of the discovery and of the various controversies held as to the origin, as well as the uses, of the galvanic current. There are many good illustrations amongst the 65 which embellish the manual and tend to facilitate our grasp of the authors' explanation. The Trough battery is simply explained on p. 33, and Fig. 9 assists us materially to follow the letterpress. The Current Controller, p. 48, is also admirably illustrated, and accurate directions are given as to the use of this excellent device, by which, we are told, "the operator holds the electric current completely under his control." The minutest dose can be given, and it can be increased or diminished by almost imperceptible degrees. The various batteries are clearly described and drawn, from the handy portable affair, familiar to us all, to the stately cabinet battery by Otto Fleming, of Philadelphia, which, when not in use, might well be mistaken for the Lady'sDavenport, to which it has a close resemblance. Under the heading "General Neuroses" comes an excellent bit of common-sense teaching: "The underlying cause should, of course, be removed, and appropriate hygienic and medical treatment supplement the electrical," and we think it would be wise to add that electrical treatment should, without any exception, be regarded wholly and solely as supplementary to the proper medical treatment and under the supervision of the qualified medical practitioner.

* "The Naulahka." By Rudyard Kipling and Wolcott Balestier. Pub.: William Heinemann, Bedford Street, W.C.

† Essentials of Medical Electricity. By D. D. Stewart, M.D., and E. S. Lawrence, M.D. Pub: W. B. Saunders, 913, Walnut Street, Philadelphia. Price $1.

### Some Thoughts about Holidays.

(From a Correspondent.)

The fashion of a regular summer holiday, which has become so general of late, is justified and, indeed, made necessary by the high pressure rate of most lives. It is not easy to restrict the work of the day ; it would be rational, indeed, to do so, and by an even, continued effort, with few breaks, we should probably accomplish more on the whole than we do now by our intense exertions, broken by the illness they induce or the necessity of running away every now and then to avoid a breakdown. Individuals, however, can do little to resist fashion or custom : happy those who grasp the situation, and in mind rise above the entanglement, while lending all their strength to lift the common burden.

Hospital nurses are among those who most need a summer holiday, and who most regularly take it. We who are in the secret of hospital life know how little it is possible there to regulate the expenditure of strength, even where the hours on duty are not unreasonable ; that is, for those who have the true vocation ; we will readily allow there are hospital workers who have not that vocation, and who working either remissly, unskilfully, or with only mechanical correctness, leave a heavy share for those who are willing and fit Willing and fit nurses ! Whose soul does not warm to them that has either experienced or seen their ministrations to their poor broken forlorn charges ?

One after another of the dear faces comes before me, while instances of their devotion, their labours, their suffering, their death, crowd upon my remembrance. Their work is only measured by their patients' needs, and their own utmost strength to meet them. When holiday time comes to these dutiful souls, the manner of spending it will seldom be an open choice. The purse has a good deal to say to it ; the family circumstances may decide it ; the possibility of an acceptable companionship is a chief consideration ; and altogether the holiday will be like most of our conditions rather shaped for us than by us. What we want is the simple heart to accept what comes to us, and to make the best of it. Sister Clemence puts away her visions of Switzerland and goes home, because her visit is the great event of the year to her widowed mother. For her reward she finds a solace in the atmosphere of that mother's love which refreshes every fibre of her wearied nature. She may sigh a little after the freshness of mountain breezes, but she will insist that the suburban air is nearly as bracing.

Nurse Constance perhaps spends one of her precious weeks in a midland town, having a feast of little nephews and nieces—healthy babies, merry and fascinating in healthy wise—and she will spend the rest of the time at the seaside with a sister engaged in teaching, whose holidays occur opportunely.

One or two of our number will go yachting or to the Scotch moors, dipping into a familiar great world, from which they will come back with renewed affection for their work. The cottage, the farm, the rectory, the castle will all claim their members from the hospital corps. A few will go off on a chosen route with a chosen friend. By and bye as the party gathers again there will be lively talk at table and in recreation hours, and much comparing of notes of the various experiences. Those who have been far afield are, perhaps, a little sorry for those who have made but a short flight. It is exhilarating to be sure to form one of a cavalcade that starts early on a brilliant morning from a hotel in the Rhone Valley and goes up the wonderful way to Zermatt. Happily the bright moments are those that return to the memory ; for people out of training, heat, weariness, and aching make no small part of each day's expedition in the mountains ; but this is hardly remembered afterwards, while the grand snowy peaks and the lovely sketches of pasture through which we ascend towards them make pictures always visible to us when we will turn our mental eyes that way.

We are interested in Alpine grandeurs and yachting adventures but our mind dwells still more fondly on familiar scenes. We have not travelled far but we have found a spangled meadow level with the shore, and lying there, lulled by the lapping of the waves, we have listened to the lark's joyous song and, watched his soaring till we lose him in the dazzling light of the sky. The sky with its gossamer clouds, "meet pavement for an angel's glorious march." And we say that earth and sea and sky are beautiful there as heart could wish.

Or we have been among the Border hills, where the burn sings its lullaby as it threads its way through bracken and heather down to its little valley fragrant with clover. If you have not been in that green pastoral country, and would like to dream you have been there, read dear Dr. John Brown's "Minchmoor," in the third volume of his "Horæ Subsescivæ," with its quotation :

> " And what saw ye there
> At the bush aboon Traquair ;
> Or what did ye hear that was worth your heed ?
> I heard the cushies croon
> Through the gowden afternoon,
> And the Quair burn singing doon to the vale o' Tweed."

"Matron's" last injunction, in saying good-bye, is, to forget all about the hospital. But she does not quite mean it, any more than nurse means her assent. And so it happens that the lambs recall the children, and, if a wish could accomplish it, poor little pale patient Johnnie and Lily, and thousands more, would be lying among the clover, sharing with us the festival of nature at her best ; and as they cannot come to us the heart quickens with the desire to go back to them, and to take them in our renewed strength and peace some of the sunshine and fragrance and sweet sights and sounds which have so delighted us. The waving grass we pluck for them will soon lose its grace, but the harvest gathered in the thankful heart will show its sustaining power in many a battle to relieve the pain of our stricken ones, and to cheer them in the accumulations of their trouble, and in the patient bearing of whatever toil and dreariness may lie between us and another holiday. A. L. P.

### Appointments.

[It is requested that successful candidates will send a copy of their applications and testimonials, with date of election, to The Editor, The Lodge, Porchester Square, W.]

Cumberland Infirmary, Carlisle.—Miss Harriete E. G. Hamilton has been appointed Matron at this infirmary. Miss Hamilton was trained by the Nightingale School at St. Thomas's Hospital, and has been "Sister" at the same hospital for four years.

Suva Hospital, Fiji.—Miss Frances Webster-Wedderburn has been appointed by the Colonial Government Matron of this hospital. Miss Wedderburn was trained by the Nightingale School at St. Thomas's Hospital, being there three years altogether. She then went as Night Superintendent to the Royal Infirmary at Derby, and remained there till ordered to take a long rest. Since then she has taken five weeks' Matron's duties at Hemel Hempstead, and has worked five weeks with the All Saints' Sisters at Cowley. Miss Wedderburn sails on September 16th.

None of the inhabitants of Dublin will regret Lady Zetland's departure more than the nurses, to whom she has ever been such a good and hospitable friend.

### The Little Sisters of the Assumption.

A friend of the Little Sisters kindly sends us the following letter :—

It will perhaps interest you to learn some further details of the work of the "Little Sisters of the Assumption," who nurse the sick poor in their own homes, and of whose origin and aims you published a short account two summers ago. I know well that every branch of nursing, and especially all that has to do with the care of the sick poor, is always of interest to your readers, so I give myself the pleasure of writing some of the particulars of this work.

Probably the previous account said that it is a young congregation, and still under the immediate superintendence of its founder, the Père Pernet, a priest of the Order of the Augustinian Fathers of the Assumption. The principal end in view in all the work of the Little Sisters is to restore the family life in the homes of the working classes. In France, as in England, this want of all true home life in the dwellings of the poor is the main cause of all their evils ; the father is at the club or the public-house, the mother is perhaps working away from home (though this is chiefly incidental to the manufacturing districts of England); perhaps visiting her neighbours; perhaps toiling at home alone, not only all the day ; but all the long evenings also ; the children when they are not at school are always turned out into the street, where the most depraved corrupt the most innocent. Doctors and nurses who work amongst the working classes will recognise the justice of these assertions.

The work of the Little Sisters is to nurse the sick poor, who are too poor to pay a nurse in their own homes ; to do the housework where that is required—cook, clean, mind the babies, wash and dress the children and send them to school ; in fact, to make herself the servant of the poor in order to gain them to God. She goes in the morning, returning for the midday meal goes again in the afternoon, returning for dinner, and never accepts even so much as a glass of water. If the patient is too ill to be left at night or during the meal times, the Little Sister is replaced by another during those intervals.

And now to describe briefly the organisation of the congregation. The Mother House is in Paris at "57, Rue Violet, Grenelle," one of the poorest parts of Paris ; there are eleven branch houses in Paris five in other towns in France ; one at New York—312, First Street East ; one at Dublin—116, James Street ; and two in London—14, Wellington Road, Bow, E., and 42 and 43, St. James's Square, Notting Hill. The English and Irish postulants spend some months at the house at Notting Hill before coming here, and the American postulants at the house at New York.

Where the Little Sisters are in full working order they have affiliated to them an association of lady servants. They are ladies living in the world who undertake to give a certain portion of their time to the sick poor, nursing them and working for them with the Little Sisters and under their direction ; they are engaged not to give money directly to the poor, but to trust it to the Little Sisters, for there is always need of it, and she, Little Sister, brings whatever is most needed, and gives it on the part of the society for the love of God. They spend hours at a time in the dwellings of the poor, acquiring thus an intimate knowledge of their needs and their manner of life ; and in spending their time and money thus in their service they prove to the working classes that they are ready and anxious to help them. It is to be hoped that this work as it extends further and further will do much to break down the barrier that custom and misunderstanding have erected between the different classes of society.

Here in Paris, and in various other places, there are also confraternities of men, composed of old patients and the husbands of old patients ; they are working men, and the object of the "Confraternity of our Lady of Salvation" is to aid and oblige the members to fulfil the duties of a husband and father in their families. They must bring all their earnings home to their wives ; watch over their children, and see that they are brought up religiously and well ; where circumstances oblige the children to be taught at a Government school, to see that they are sent to Catechism ; to have no literature in the house contrary to faith or morals, nor any bad pictures either ; and, of course, to fulfil the obligations of the Catholic Church, such as Mass on Sundays, abstinence on Fridays, &c. At the head of this society there are gentlemen who speak at the conferences on the topics of the day, social and religious, and who each overlook ten families of the members, calling on them in their homes at any time, and seeing that the regulations are faithfully observed. The probation before joining this society is rather strict, sometimes the men wait even a year ; still, there are now about 500 members in Paris only. Here and in many other towns also there is another branch society, the "Daughters of S. Monica," composed of the wives of those working men bound together for mutual aid and encouragement in the fulfilment of the duties of a Christian wife and mother. They have frequent meetings at the Convents, where the Little Sisters, with the assistance of the lady servants, address them on topics interesting and useful, and are ready to hear anything they care to say, and to advise and help them where they have need of aid.

And now, having described the exterior work, let me say something of the formation of the workers. If, after a period of never less than three months, a postulant is found fitted for the vocation, she receives the habit and begins her novitiate of two years. The first year is spent in the formation of the religious life—prayer, study, and household work are the employments of the day. As every Little Sister is sure, one day, to find herself amongst people prejudiced against religion, against authority and social order, it is necessary that she should be able to refute those prejudices, and that is only possible by the study of Church history, and a thorough practical knowledge of the doctrines of the Church, and the reason of her ceremonies; therefore, the first year novices spend much of their time in study. During the second year the novices go to the sick with an experienced Sister, who trains them in the nursing and other work in the homes of the poor. The Little Sisters are bound to an absolute obedience to the orders of the doctor, and an entire loyalty to him in all ways. At the end of two years the novice may be professed, and sent to any part of the world where there are houses of the congregation.

Such are the aims, and such is the work of the Little Sisters. To nurse the sick poor, to restore the family life in their dwellings, to combat sin and unbelief, to bring to the dying the aids and consolations of religion. You, who hear so much of the misery, physical, and often moral, of the sick poor will be interested, I believe, in this brief notice of a work that exists only for their aid. Again I thank you for your unfailing kindness on all occasions.

### Royal Berks Hospital, Reading.

The nurses and probationers of this hospital have been entertained at Englefield House by the kind invitation of Mr. and Mrs. Benyon, who sent brakes for them on two different days so that all the nursing staff should have an opportunity of enjoying the visit. The head gardener took them all over the beautiful gardens and hot-houses, and afterwards they were allowed to make a complete inspection of the fine old Tudor mansion with its many historical associations and treasures. Mrs. and Miss Benyon received them all in the library, and gave them tea in the Long Gallery, after which the conservatories and park were visited.

### A Long Day.

The longest day I ever spent in my life was a bright and sunny one in last July. An East End parson, one of the hard workers in that world of toilers, had retained my services to help his wife and himself on the occasion of a "day in the country" with his night-school boys. I am glad to think that the class was but recently organized, for otherwise the dicipline of those lads left much still to be desired!

We started early and got our cargo of ragged humanity safely bestowed in a railway carriage, which had been reserved for the party; such a collection of eager-eyed, sharp-faced, poorly-clad creatures, restless and rude, but wonderfully good-tempered, and showing unexpected consideration for the youngest boy of the party, and also for one poor little cripple "crooked Jim" to whom a "window seat" was voluntarily yielded by the roughest member of the tribe, he having previously obtained possession of it by a rather alarming display of brute force. After an hour's journey we reached our station, and, how can I describe it? Before we three anxious guardians could foresee the danger, much less escape it, those fifty wild boys had descended with a rush on to the platform, had made a raid on the station master's well-kept garden and, in a second, every rose was stripped from the trees. It was a dreadful moment, no possible after action could undo the mischief, all the penalties of the law, including solitary confinement and a frugal diet of "skilly" flashed across my mind, but ere I found words to apologise, that worthy and stately official bore hastily down upon us and his pleasant voice sounded in my ear, "Why God bless ye, ma'am! don't ye take it so to heart; poor little chaps, happen they never see such flowers growing so free 'afore," and with a calm and smiling face the good man helped us to pack our charges into the waiting waggons, but I may as well add here that when, on our return in the evening those untamed lads again invaded the platform, they found that each rose-bush on the opposite side, which had escaped them in the morning, was now under the special charge of a porter or other upholder of authority, and so their unholy design to annex the rest of the blossoms was luckily frustrated.

The drive from the station was by no means uneventful, for first one and then another child managed to delay our progress by scrambling out and darting into an enticing wood which bordered the road. And when this temptation was left behind a practical joker contrived to throw a neighbour's cap into a pond, from which it was eventually rescued after a good deal of time, trouble, and ingenuity had been consumed.

At last our destination was reached, and our gracious hostess awaited us, in front of a tent where a substantial cold lunch was prepared. We swiftly alighted, and somewhat anxiously marshalled our party, bidding them remember that they must reply civilly to the welcome they were receiving, and reminding them that this was the moment they had been previously prepared for, when they were expected "to do credit to themselves and their teachers." Oh, would they remember, would they condescend to behave, for this short interval like civilised beings?

The question was speedily settled by a wild shriek from the biggest of the boys, which was rapidly followed by a stampede of them all. Across the trim lawns and over the gay flower-beds, straight on they sped to the gleaming lake beyond which seemed to attract them as the magnet does the needle. There it lay, flashing like silver in the sunshine, as we hastily followed our unruly flock, only partially re-assured by our hostess' merry voice calling after us "never mind, it is not at all deep" but ere we gained the water's edge what a sight met our eyes. Each little lad had shed every bit of his ragged clothing helter-skelter on the path, and dashed into the smiling water, and ere the return to dry land was made, all the water lilies, the pride of the pond, were torn up by those eager little ravagers. The beautiful white and yellow flowers, with their shining leaves and the long tough stems which had yielded unwillingly to the rough strain made upon them all, all were drawn away in triumph by the small and dripping guests.

When, this episode over, and the ragged garments donned again, the youngsters were safely collected in the tent, they showed themselves well able to do full justice to the pleasures of the dinner table, and if their mode of eating was somewhat of a wolfish character, it was hardly to be wondered at when we thought of the uncouth surroundings from which they had been caught away to enjoy this one day in a veritable country paradise.

A tendency to pocket some of the delicacies for friends at home had to be discountenanced, but a parting gift of oranges and huge wedges of cake made up handsomely for the momentary disappointment. For the adults who went with them, however, it was truly "a long day."

### Notes and Queries.

To Correspondents.—1. Questions or answers may be written on post-cards. 2. Advertisements in disguise are inadmissible. 3. In answering a query please quote the number. 4. A private answer can only be sent in urgent cases, and then a stamped addressed envelope must be enclosed. 5. Every communication must be accompanied by the writer's full name and address, not necessarily for publication. 6. Correspondents are requested to help their fellow nurses by answering such queries as they can.

### Queries.

Can anybody tell me of a really good nurses' home or holiday home at Scarborough.—*M. R.*

### Answers.

*Miss Blake Forster.*—Consult a surgeon. We never prescribe. This is a rule to which no exceptions can be made.

*Folkestone.*—Certainly, advertise at once; it's no use your putting it off till the last moment.

*Midwifery Books, Brymbo.*—Either "Playfair's Midwifery" or "Barnes' Manual of Midwifery" would be very useful. You might get a second-hand copy through an advertisement.

*Caro.*—The agency you name is very fairly good, or you could advertise; but as you have only just that limited time to work in, perhaps you had better go to the agency; it is such a chance unless you know several London doctors.

*Further Training, Dorothy.*—There are about twenty applications for each vacancy at Edinburgh Royal Infirmary, and a personal interview is necessary. Bath Royal United Hospital, Cumberland Infirmary, Carlisle; Nottingham General Hospital.

*Paralytic.*—We will ask in our columns, and let you know as soon as possible.

*Matron Dispenser, P.C.*—A Matron Dispenser would be quite suitable for your small hospital, and your best plan is to advertise your requirements.

*Victoria.*—You must send name and address. In the future we shall make no exception to this rule.

*A Nurse of Some Years' Standing.*—Your case is no doubt similar to that of many others, but we must congratulate you in not having your name on in company with those cases you mention. We do not publish your letter, because just now it is better not. Go and see Mrs. Nichol, at the Midwives' Institute, and tell her about it. We are much obliged to you for telling us about it, and should like to know your friend's name on whose recommendation you applied.

*Alexandria.*—Communications received; many thanks.

*Sister E., Colchester.*—We have inserted your letter, and therefore cannot return it.

*N. P.*—Yes; three years in a good general hospital. We cannot send answers by post, unless the matter is really urgent or private, or the answer too long for our columns. Even stamps will not tempt us in the future; our correspondence, as it is, is very large, and ever increasing.

*Old Subscriber.*—Do not take any notice; allegations are easily made, and if they are true, both you and all of us would be ready to see justice done; but there are people who live for nothing but vilifying other people and things. Let the date, name, and all particulars be forthcoming first. They have a family likeness to some other allegations equally untrue.

## Figures and Tables

**Figure f1:**